# Different Diet Energy Levels Alter Body Condition, Glucolipid Metabolism, Fecal Microbiota and Metabolites in Adult Beagle Dogs

**DOI:** 10.3390/metabo13040554

**Published:** 2023-04-13

**Authors:** Haoran Sun, Qiaoru Zhang, Chao Xu, Aipeng Mao, Hui Zhao, Miao Chen, Weili Sun, Guangyu Li, Tietao Zhang

**Affiliations:** 1 Institute of Special Animal and Plant Sciences, Chinese Academy of Agricultural Sciences, Changchun 130112, China; solomonsunhr@outlook.com (H.S.); xuchao@caas.cn (C.X.); zhaohui02@caas.cn (H.Z.); sunweili@caas.cn (W.S.); 2 College of Animal Science and Technology, Hebei Normal University of Science and Technology, Qinhuangdao 066004, China; 3 College of Animal Science and Technology, Qingdao Agricultural University, Qingdao 266000, China

**Keywords:** diet energy, pet food, beagle dog, obesity, glucolipid metabolism, fecal microbiota, metabolomics, SCFAs, bile acids

## Abstract

Diet energy is a key component of pet food, but it is usually ignored during pet food development and pet owners also have limited knowledge of its importance. This study aimed to explore the effect of diet energy on the body condition, glucolipid metabolism, fecal microbiota and metabolites of adult beagles and analyze the relation between diet and host and gut microbiota. Eighteen healthy adult neutered male beagles were selected and randomly divided into three groups. Diets were formulated with three metabolizable energy (ME) levels: the low-energy (Le) group consumed a diet of 13.88 MJ/kg ME; the medium-energy (Me) group consumed a diet of 15.04 MJ/kg ME; and the high-energy (He) group consumed a diet of 17.05 MJ/kg ME. Moreover, the protein content of all these three diets was 29%. The experiment lasted 10 weeks, with a two-week acclimation period and an eight-week test phase. Body weight, body condition score (BCS), muscle condition score (MCS) and body fat index (BFI) decreased in the Le group, and the changes in these factors in the Le group were significantly higher than in the other groups (*p* < 0.05). The serum glucose and lipid levels of the Le and He groups changed over time (*p* < 0.05), but those of the Me group were stable (*p* > 0.05). The fecal pH of the Le and He groups decreased at the end of the trial (*p* < 0.05) and we found that the profiles of short-chain fatty acids (SCFAs) and bile acids (BAs) changed greatly, especially secondary BAs (*p* < 0.05). As SCFAs and secondary BAs are metabolites of the gut microbiota, the fecal microbiota was also measured. Fecal 16S rRNA gene sequencing found that the Me group had higher α-diversity indices (*p* < 0.05). The Me group had notably higher levels of gut probiotics, such as *Faecalibacterium prausnitzii*, *Bacteroides plebeius* and *Blautia producta* (*p* < 0.05). The diet–host–fecal microbiota interactions were determined by network analysis, and fecal metabolites may help to determine the best physical condition of dogs, assisting pet food development. Overall, feeding dogs low- or high-energy diets was harmful for glucostasis and promoted the relative abundance of pathogenic bacteria in the gut, while a medium-energy diet maintained an ideal body condition. We concluded that dogs that are fed a low-energy diet for an extended period may become lean and lose muscle mass, but diets with low energy levels and 29% protein may not supply enough protein for dogs losing weight.

## 1. Introduction

Obesity is the most common nutritional disease in domestic dogs and cats [1,2,3] and there are a variety of commercial veterinary diets in the pet food market and animal practice targeting this issue. The energy levels and nutritional compositions of veterinary diets for weight loss vary considerably. The metabolizable energy (ME) level ranges from 11.41 to 16.22 MJ/kg for dry diets and from 2.23 to 4.19 MJ/kg for canned diets [4]. The protein content ranges from 24.5 to 33.82 g/MJ ME, fat from 6.88 to 10.70 g/MJ ME [5] and dry mater (DM)-based total dietary fiber from 7.7 to 42.6% [6]. Selecting an effective weight loss diet for a pet is therefore difficult for pet owners and veterinarians [7]. The core of a therapeutic weight management diet is to restrict energy intake and to avoid potential nutritional deficiencies that may occur with energy restriction [8,9]. The diversity of the nutritional composition of weight loss diets may result from the limited knowledge of the interactions between diet energy and body metabolism [10,11,12].

Recent research on canine obesity has focused on validating the results of weight loss diets and the metabolism characteristics of high-fat-induced obesity. In two studies, obese or overweight dogs with restricted energy intake showed decreased body weight and body condition scores (BCS) over two months [2,13]. Methods of energy restriction include changing the macronutrient composition, such as high-protein high-fiber diets [14,15,16,17] or high-protein medium-carbohydrate diets [18], and the use of nutritional additives [19,20] or high fiber or polysaccharides [21,22,23,24]. During high-fat-diet-induced canine obesity modeling, research showed changes in glucose homeostasis, the gut microbiota and the metabolome [25,26,27,28,29].

Carbohydrates and fat are two of the main energy substances that are important in losing weight, and there has been some research in recent years on the effects of energy substances on glucolipid metabolism and the fecal microbiota [30,31]. The interactions between diet, host and fecal microbiota are also poorly explored in canine nutrition research. If the diet energy level changes, then the host and gut microbiota metabolism will also change and have an interaction. This study therefore aimed to investigate the long-term effects of the diet energy level on body condition, glucolipid metabolism, fecal microbiota and metabolites in beagles, reveal its possible health effects and interactions and lay a foundation for developing canine weight loss diets.

## 2. Materials and Methods

The experimental procedures for animal trials were approved by the Animal Ethics Committee of the Chinese Academy of Agricultural Sciences and performed according to the guidelines for animal experiments set by the National Institute of Animal Health.

### 2.1. Animals and Experimental Design

The experiment lasted 10 weeks, with a two-week acclimation period and an eight-week test phase, and was conducted at the Companion Animal Test Base, in Zuojia, Jilin, China (125°19′50″ N 43°49′46″ E; elevation 313 m). The average temperature was 21.6 °C, ranging from 12 °C to 29 °C, and the average humidity was 63%, ranging from 44% to 77% in the test phase.

Eighteen healthy adult neutered male beagles aged 1.33 ± 0.47 years, with mean body weight of 12.52 ± 2.09 kg and body condition scores (BCS) of 3.89 ± 0.99, were randomly assigned to three groups, based on the test diet. Individual groups were maintained in the same semi-closed paddock (5 × 7 m), and every dog was placed in an individual 1.2 × 1 × 1 m cage when feeding. All dogs were vaccinated and dewormed before the study.

The diets were prepared as dry kibble and were formulated to meet the nutrient requirements of adult dogs at maintenance [32], with three different energy levels, shown in Table 1. All dogs were fed the same medium-energy diet with 15.04 MJ/kg ME in the two-week acclimation period to remove the effects of the previous dog food and then randomly allotted to one of the three dry diet groups: the low-energy diet (Le) group, with dietary ME of 13.88 MJ/kg; the medium-energy diet (Me) group, with dietary ME of 15.04 MJ/kg; and the high-energy (He) group, with dietary ME of 17.05 MJ/kg. The three diets were designed with different levels of fat and nitrogen-free extracts (NEF), but the same content of protein.

All dogs were individually fed 32 g/BW kg/d twice a day at 9 a.m. and 4 p.m. and had free access to fresh water. We performed multiple measurements: fasting serum biochemistry analysis; fasting weighing; BCS (using a nine-point system, where 1–3 indicates a less than ideal body condition, 4–5 indicates an ideal body condition and 6–9 indicates an overly ideal body condition) [33]; muscle condition score (MCS) (using a four-point system, where a score of four means normal muscle mass, three indicates mild muscle loss, two is moderate muscle loss and one is severe muscle loss) [34]; body fat index (BFI) (using a BFI risk chart, where a score of 20 (16–25% body fat) = low risk, 30 (26–35% body fat) = moderate risk, 40 (36–45% body fat) = high risk, 50 (46–55% body fat) = serious risk, 60 (56–65% body fat) = severe risk, 70 (66–75% body fat) = extreme risk) [35]; and fecal pH. On day zero of the trial (T0) and on the last day of the trial (T8), all these measurements were analyzed, and fresh feces were collected from the rectum. Fecal samples were kept at −80 °C. During the trial, weighing was carried out once every two weeks and serum biochemistry analysis once every four weeks.

### 2.2. Serum Biochemistry Analysis

Blood samples were taken on T0, T4 and T8 after overnight fasting. After centrifugation, the supernatants were collected as serum samples to measure glucose (GLU), total cholesterol (CHO), triglyceride (TG), low-density lipoprotein cholesterol (LDL-C) and high-density lipoprotein cholesterol (HDL-C) using an automatic Vitalab Selectra E biochemical analyzer (Vital Scientific, Spankeren, The Netherlands).

### 2.3. Fecal pH Analysis

On T0 and T8, fresh fecal pH was immediately measured after 10% fecal suspension (*w*/*v*) in ultrapure water with a PHS-3C pH meter (INESA Scientific Instrument Co., Ltd., Shanghai, China).

### 2.4. Content Analysis of SCFAs

Fecal samples collected at T0 and T8 were homogenated for 1 min with 500 μL of water and 100 mg of glass beads and then centrifuged at 4 °C for 10 min at 12,000 rpm. Then, 200 μL supernatant was extracted with 100 μL of 15% phosphoric acid and 20 μL of 375 μg/mL 4-methyl valeric acid solution as the internal standard and 280 μL ether. The samples were then centrifuged at 4 °C for 10 min at 12,000 rpm after vortexing for 1 min, and the supernatant was transferred into the vial before gas chromatography–mass spectrometer (GC-MS) analysis [36,37,38]. SCFA content was analyzed at Shanghai Personal Biotechnology Co., Ltd. (Shanghai, China).

All target standards were purchased from Sigma-Aldrich (St. Louis, MO, USA). The GC analysis was performed on a trace 1310 gas chromatograph (Thermo Fisher Scientific, Waltham, MA, USA). The GC was fitted with a HP-Innowax 30 m × 0.25 mm ID × 0.25 μm capillary column (Agilent, Santa Clara, CA, USA) and helium was used as the carrier gas at 1 mL/min. The injection was performed in split mode at 10:1, with an injection volume of 1 μL and an injector temperature of 250 °C. The temperatures of the ion source and MS transfer line were 300 °C and 250 °C, respectively. The column temperature was programmed to increase from an initial temperature of 90 °C, followed by an increase to 120 °C at 10 °C per min, then to 150 °C at 5 °C per min and finally to 250 °C at 25 °C per min, which was maintained for 2 min. Mass spectrometric detection of metabolites was performed on an ISQ 7000 (Thermo Fisher Scientific, Waltham, MA, USA) in electron impact ionization mode. Single ion monitoring (SIM) mode was used with the electron energy of 70 eV. Six SCFAs were measured, including acetic acid, propionic acid, isobutyric acid, butyric acid, isovaleric acid and valeric acid.

### 2.5. Content Analysis of BAs

The protocol for quantifying bile acids was adapted and modified from the methods previously described by Bhargava et al. [39]. Fifty mg samples were extracted in 400 μL of methanol at −20 °C, with 100 mg of glass beads, vortexed for 60 s. They were then placed into a tissue grinder at 55 Hz for 1 min and this was repeated at least twice. The sample was next sonicated for 30 min at room temperature, centrifuged at 12,000 rpm and 4 °C for 10 min, and 300 μL of the supernatant was mixed with 600 μL of water and vortexed for 30 s. Then, 10 μL supernatant was added to 30% methanol and diluted 10 times. The supernatant was filtered through a 0.22 μm membrane and the filtrate was added to the LC-MS bottle. BA content was analyzed at Shanghai Personal Biotechnology Co., Ltd. (Shanghai, China).

The target standards were purchased from Sigma-Aldrich (Shanghai, China), Macklin (Shanghai, China), Shyuanye (Shanghai, China), Toronto Research Chemicals (Toronto, ON, Canada), Zzsrm (Shanghai, China) and the National Institutes for Food and Drug Control (Beijing, China). The supernatant was analyzed by gas chromatography and mass spectrometry according to the methods described by Hu et al. [40] and Yang et al. [41]. A 2.1 × 100 mm, 1.7 μm Acquity UPLC^®^ BEH C18 column (Waters, Milford, MA, USA) was used, the injection volume was 5 μL, the column temperature was 40 °C and the mobile phase was A-0.01% formic acid water and B-acetonitrile. The gradient elution conditions were 0–9 min, 30% B, 9–14 min, 30–36% B, at a flow rate of 0.3 mL/min, 14–18 min, 36–38% B; 18–24 min, 38% to 50% B; 24–32 min, 50–75% B; 32 to 33 min, 75–90% B; 33–35.5 min and 90–30% B at a flow rate of 0.25 mL/min. The mass spectrophotometer used an electrospray ionization (ESI) source, in negative ionization mode. The ion source temperature was 500 °C, the ion source voltage was −4500 V, the collision gas was 6 psi, the curtain gas was 30 psi and the atomizing gas and auxiliary gas were both 50 psi. Scans were performed using multiple reaction monitoring (MRM). Thirty-nine bile acids were measured, including deoxycholic acid (DCA), ursocholic acid (UCA), allocholic acid (ACA), glycohyodeoxycholic acid (GHDCA), chenodeoxycholic acid-3-β-D-glucuronide (CDCA-G), dehydrocholic acid (DHCA), taurodeoxycholic acid sodium salt (TDCA), taurochenodeoxycholic acid (TCDCA), lithocholic acid (LCA), chenodeoxycholic acid (CDCA), ursodeoxycholic acid (UDCA), cholic acid (CA), glycochenodeoxycholic acid sodium salt (GCDCA), glycodeoxycholic acid sodium salt, (GDCA), sodium glycocholate hydrate (GCA), taurocholic acid sodium salt (TCA), taurohyodeoxycholic acid sodium salt (THDCA), allolithocholic acid (isoLCA), isolithocholic acid (alloLCA), 23-nordeoxycholic acid (NorDCA), 6-ketolithocholic acid acetate, (6-ketoLCA), 12-ketolithocholic acid (12-ketoLCA), 7-ketolithocholic acid (7-ketoLCA), 3β-ursodeoxycholic acid (β-UDCA), hyodeoxycholic acid, (HDCA), norcholic acid (NorCA), 7,12-diketolithocholic acid (7,12-diketoLCA), 6,7-diketolithocholic acid (6,7-diketoLCA), α-muricholic acid (α-MCA), β-muricholic acid (β-MCA), 3β-cholic acid (β-CA), glycolithocholic acid sodium salt (GLCA), glycoursodeoxycholic acid (GUDCA), lithocholic acid 3-sulfate sodium salt (LCA-3S), taurolithocholic acid sodium salt (TLCA), tauro-α-muricholic acid sodium salt (T-α-MCA), taurohyocholic acid sodium salt (THCA), tauro-β-muricholic acid sodium salt (T-β-MCA) and tauroursodeoxycholic acid (TUDCA).

### 2.6. Microbial Analysis

#### 2.6.1. DNA Extraction, Amplification and Sequencing

A 0.2 g fecal sample collected at T0 and T8 was stored in dry ice and sent to Shanghai Personal Biotechnology Co., Ltd. for 16S rRNA detection of the intestinal microbiome. Total genomic DNA samples were extracted using the M5635-02 soil DNA kit (Omega Bio-Tek, Norcross, GA, USA), following the manufacturer’s instructions, and stored at −20 °C before further analysis. The quantity and quality of extracted DNA were measured using a NanoDrop NC2000 spectrophotometer (Thermo Fisher Scientific, Waltham, MA, USA) and agarose gel electrophoresis, respectively.

Polymerase chain reaction (PCR) amplification of the bacterial 16S rRNA genes V3–V4 region was performed using the primers 5′-AYTGGGYDTAAAGNG-3′ (520F) and 5′-TACNVGGGTATCTAATCC-3′ (802R). Paired-end sequencing was used in the Illumina NovaSeq platform with the NovaSeq 6000 SP Reagent Kit (San Diego, CA, USA) at Shanghai Personal Biotechnology Co., Ltd. (Shanghai, China).

#### 2.6.2. Bioinformatics Analysis

Microbiome bioinformatics was performed with QIIME2(2019.4) [42] with slight modifications according to the official tutorials https://docs.qiime2.org/2019.4/tutorials/ (accessed on 1 March 2023). Sequences were then quality-filtered, denoised, merged and chimera-removed using the DADA2 plugin [43]. Non-singleton amplicon sequence variants (ASVs) were aligned with MAFFT [44] and used to construct a phylogeny with FastTree2 [45]. Alpha-diversity and beta-diversity metrics were estimated using the diversity plugin with samples rarefied to 97% sequences per sample. Taxonomy was assigned to ASVs using the classify-sklearn naïve Bayes taxonomy classifier in the feature-classifier plugin [46] against the SILVA Release 132 Database [47].

Sequence data analyses were mainly performed using QIIME2 (2019.4) and R packages (v3.2.0). A heat map showed the top 20 genera in terms of relative abundance, and it was constructed using the R language and PheatMap package. A Venn diagram was generated to visualize the shared and unique ASVs among samples or groups using the R package VennDiagram, based on the occurrence of ASVs across groups regardless of their relative abundance [48]. Linear discriminant analysis effect size (LEfSe) was performed to detect differentially abundant taxa across groups using the default parameters [49]. Random forest analysis was applied to discriminate the samples from different groups using QIIME2 with default settings, and the target variable was diet [50,51]. Microbial functions were predicted using the phylogenetic investigation of communities by reconstruction of unobserved states (PICRUSt2) [52] based on the Kyoto Encyclopedia of Genes and Genomes (KEGG) at https://www.kegg.jp (accessed on 1 March 2023).

### 2.7. Statistical Analysis

Statistical analysis was performed using GraphPad Prism 9.5 (GraphPad, San Diego, CA). Data are presented as the mean ± SEM (standard error of mean). Statistical software SPSS 19.0 was used for one-way or two-way with time and treatment interaction analysis of variance (ANOVA) and the Turkey postmortem test was used to compare the differences between the groups with the three diet energy levels. The fixed variable was diet and the random variable was dog. *p* < 0.05 was considered significant.

## 3. Results

### 3.1. Changes in Body Weight and Body Condition of Beagles with Different Diet Energy Levels

**Figure 1:**
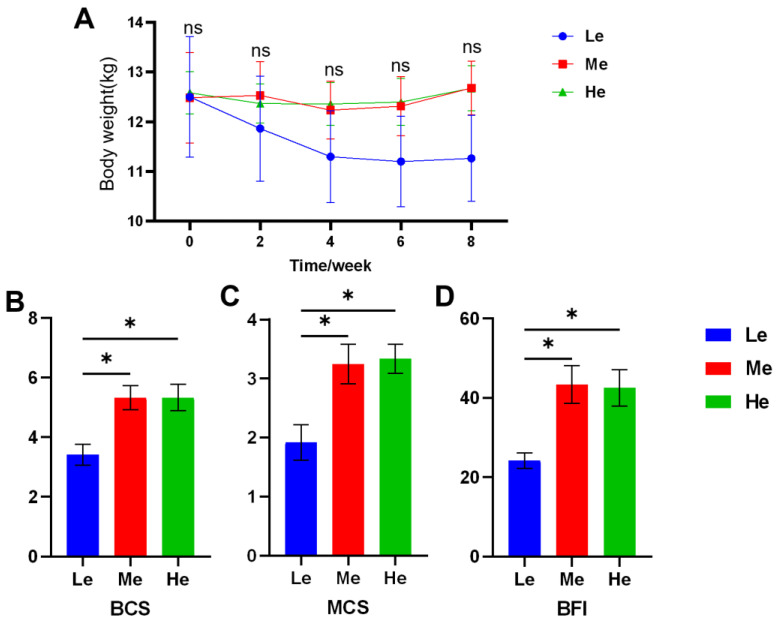
Effects of diet energy level on body weight in adult beagles (**A**); body condition score (BCS), muscle condition score (MCS) and body fat index (BFI) at the end of the experiment (**B**–**D**). Data are shown as mean ± SEM. * *p* < 0.05.

During the eight-week experiment, the body weight (BW) of dogs was as shown in Figure 1A and Table 2. As the BW of the Le group decreased rapidly, the average daily gain (ADG) was −44.05 g/d, and the other two groups were significantly higher than Le (*p* < 0.05) and over zero, as seen in Table 3. The BCS, MCS and BFI of the Le group at T8 were significantly lower than those of the Me and He groups (*p* < 0.05), and changes in body condition items showed a linear relation with diet energy (*p* < 0.05), as seen in Figure 1B–D and Table 3.

### 3.2. Changes in Blood Glucose and Blood Lipid Levels of Beagles with Different Diet Energy Levels

As shown in Table 4, the CHO content of the He group and the TG content of the Le group in serum increased significantly (*p* < 0.05) and the serum CHO of eight beagles in the Me and He groups was more than the normal reference range in the eight-week trial, compared with only one beagle in the Le group.

The LDL-C content and the ratio in CHO rose significantly with time (*p* < 0.05), especially in the He group. The serum HDL-C content at T8 increased as the diet energy levels were increased (*p* < 0.05), and the ratio of HDL-C in CHO increased over time (*p* < 0.05).

The GLU content changed greatly over time in the Le and He groups (*p* < 0.05). In general, the diet energy levels affected the glucose and lipid metabolism of the trial beagles, with higher diet energy levels harming the serum lipid levels and medium diet energy levels assisting glucostasis.

### 3.3. Changes in Fecal pH, SCFAs and BA Levels of Beagles with Different Diet Energy Levels

**Figure 2:**
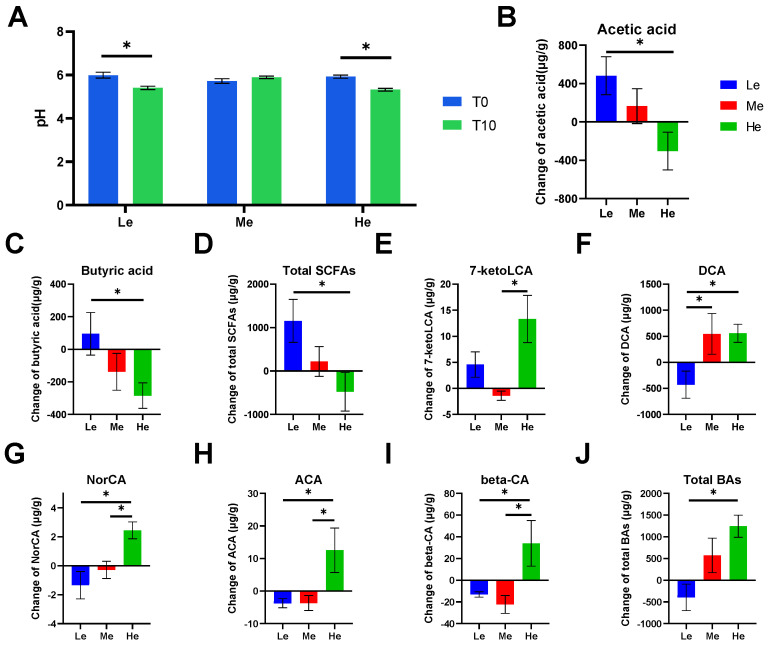
Effects of diet energy levels on fecal pH of beagles (**A**); changes in fecal acetic acid, butyric acid and total short-chain fatty acids (SCFAs) between T0 and T8 (**B**–**D**); changes in fecal 7-ketolithocholic acid (7-ketoLCA), deoxycholic acid (DCA), norcholic acid (NorCA), allocholic acid (ACA), 3β-cholic acid (β-CA) and total bile acids (BAs) between T0 and T8 (**E**–**J**). Data are shown as the mean ± SEM. * *p* < 0.05.

**Figure 3:**
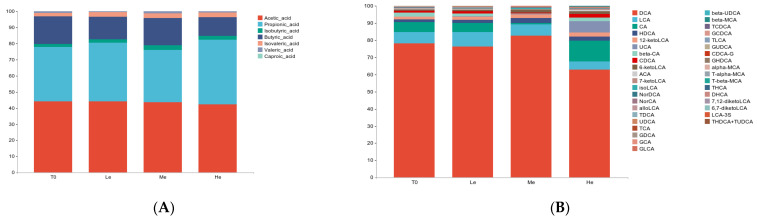
Effects of diet energy levels on fecal SCFA (**A**) and BA (**B**) proportions in adult beagles.

Feces became more acidic when beagles were fed a lower- or higher-energy diet, as the fecal pH of the Le and He groups significantly decreased by T8 (*p* < 0.05, Figure 2A). The content of SCFAs and BAs in beagle feces was analyzed and showed that different diet energy levels changed the metabolite profiles of fecal SCFAs and BAs, as shown in Figure 3A,B.

SCFAs are major end products of bacterial carbohydrate fermentation in the intestinal tracts of dogs [54]. Fecal total SCFAs increased the most in the Le group, which had the most carbohydrates, but decreased in the He group, which had the least carbohydrates (*p* < 0.05), as shown in Figure 2D. The same trend occurred for linear chain SCFAs, such as acetic acid and butyric acid (*p* < 0.05), as shown in Figure 2B,C.

Bile acids have lipid-digestive functions [55]. The changes in fecal total BAs were significantly higher in the He group compared with the Le group (*p* < 0.05) between T8 and T0, and the content of total BAs decreased in the Le group, as seen in Figure 2J. Changes in five secondary BAs in the He group, including 7-ketoLCA, DCA, NorCA, ACA and β-CA, were the highest between the three groups (*p* < 0.05), as shown in Figure 2E–I. However, changes in primary BAs, such as CDCA, CA, GCDCA, GCA and TCDCA, were not altered by diet energy levels (*p* > 0.05).

**Figure 4:**
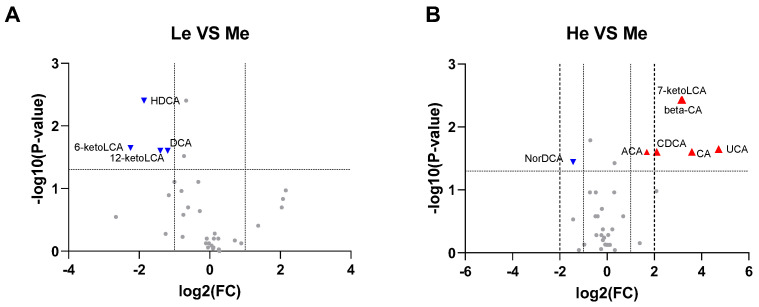
Volcano plots for Le vs. Me (**A**) and He vs. Me (**B**). Blue triangles represent significantly downregulated metabolites (log2 fold change < −1 and *p* < 0.05) whereas red triangles represent significantly upregulated metabolites (log2 fold change > 1 and *p* < 0.05).

Using all the quantitative analysis data of six SCFAs and 39 BAs at T8 to construct volcano plots, it was found that 6-ketoLCA, 12-ketoLCA, DCA and HDCA were downregulated in the Le group, while 7-ketoLCA, β-CA, CA, CDCA, UCA and ACA were upregulated and NorDCA downregulated in the He group when compared with the Me group (−1 ≥ log_2_ fold change ≥ 1 and *p* < 0.05), as seen in Figure 4A,B.

### 3.4. Changes in the Structure and Composition of Fecal Microbiota of Beagles with Different Diet Energy Levels

**Figure 5:**
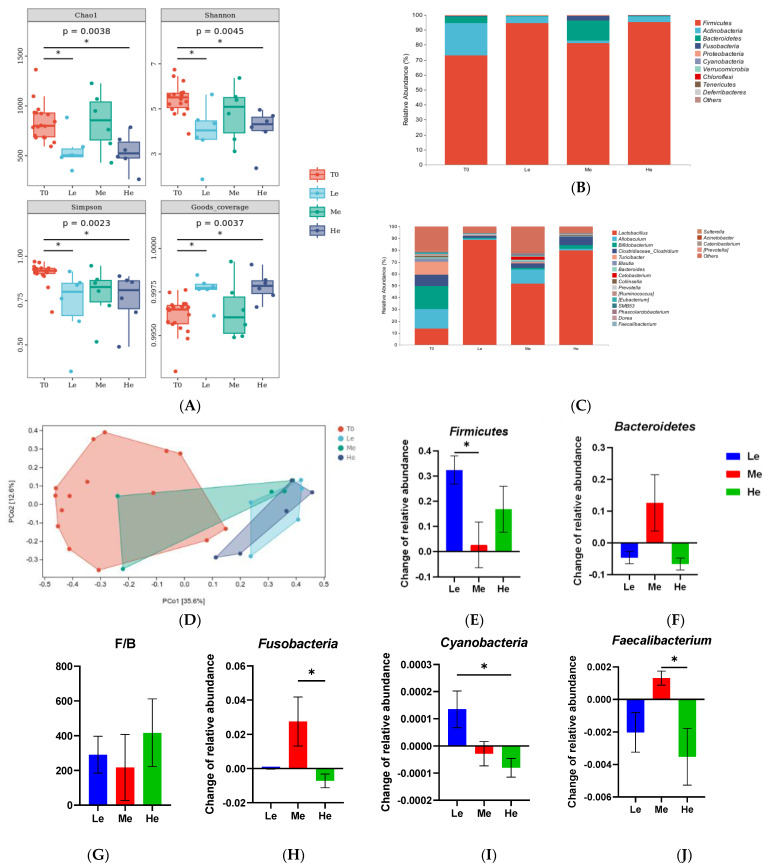
α-Diversity indices (Chao1, Shannon, Simpson and Goods coverage) of fecal microbial communities of the T0, Le, Me and He groups (**A**). The taxonomic composition of fecal microbiota at the phylum (**B**) and genus (**C**) levels of the T0, Le, Me and He groups. Principal coordinate analysis (PCoA) of β-diversity of the T0, Le, Me and He groups (**D**). Species with major differences at the phylum (**E**–**I**) and genus (**J**) levels. Data are shown as mean ± SEM. * *p* < 0.05.

As the gut microbiota ferment SCFAs and modify primary BAs to secondary BAs, the structure and composition of the fecal microbiota from the rectum were analyzed. As shown in Figure 5A, the time factor had no significant effects on the Chao1, Shannon, Simpson and Goods coverage indexes of fecal microbiota α-diversity when dogs were fed the same energy diet (*p* > 0.05). However, dogs in the Le and He groups showed lower levels of fecal microbiota α-diversity compared with the T0 group (*p* < 0.05). As seen from the results of the PCoA analysis of β-diversity in Figure 5B, the distribution ranges of the fecal microbiota in the Le and He groups were similar, but they were different from the T0 group. The PCoA of Bray–Curtis distances showed that the diet energy level could influence the gut microbiota composition and structure in dogs.

As shown in Figure 5C,D, the profiles of the phylum and genus levels in the four groups were different. At the phylum level, as shown in Figure 5C,E–I, the changes in the relative abundances of *Firmicutes* between T8 and T0 in the Le group were significantly higher than in the Me group (*p* < 0.05). The changes in *Bacteroidetes* were higher and the ratio of *Firmicutes*/*Bacteroidetes* (F/B) was lower in the Me group than that in the other groups (*p* > 0.05), but it did not show a significant difference because of the great intra-group difference. *Fusobacteria* and *Cyanobacteria* of the He group were also different from those of the other groups (*p* < 0.05). At the genus level, as seen in Figure 4D, *Faecalibacterium* had the most obvious change in abundance. The relative abundance of *Faecalibacterium* increased in the Me group while decreasing in the Le and He groups (*p* < 0.05).

### 3.5. Effects of Diet Energy Level on Fecal Microbiota and Metabolic Pathways

**Figure 6:**
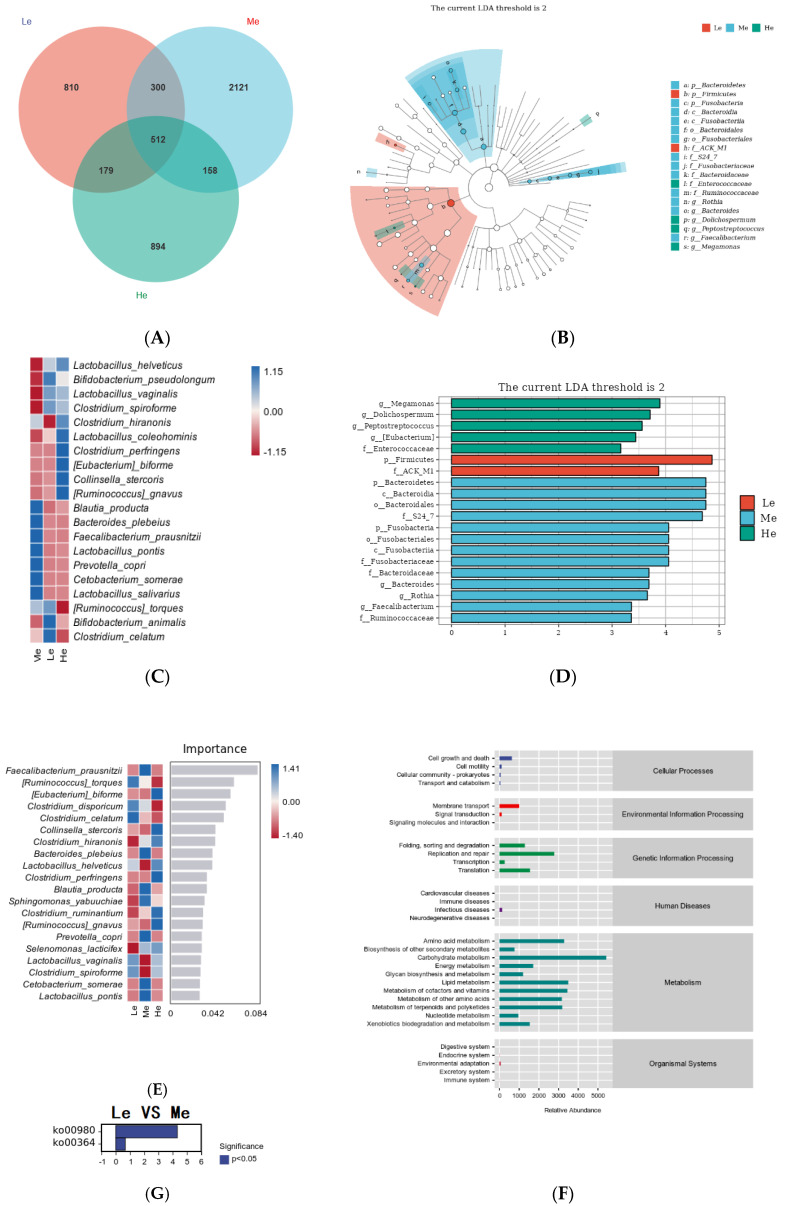
Venn diagram of the Le, Me and He groups (**A**). LEfSe analysis identified fecal bacterial biomarkers of the Le, Me and He groups (**B**,**C**). Heat map of species composition (**D**). Random forest model of species level (**E**). Metabolic pathways analysis (KEGG database) of the Le, Me and He groups (**F**). The upregulated metabolic pathways of the Le group compared with the Me group (**G**).

To further study the effects of diet energy factors on fecal microbiota without the time factor, the species differences and marker species between the three groups at T8 were analyzed. As shown in Figure 6A, 1801 amplicon sequence variants (ASVs) were obtained in the Le group, 3091 ASVs were obtained in the Me group and 1743 ASVs were obtained in the He group. Compared with the Me group, the numbers of ASVs in the Le and He groups decreased by 41.73% and 43.61%, respectively. The number of ASVs shared among the three groups was 512, the proportion of unique ASVs in the Le and He groups decreased and the numbers of unique ASVs in the two groups changed from 68.62% in the Me group to 49.98% and 51.29%, respectively.

To select the differential bacteria, differential abundant taxa were confirmed by LEfSe analysis, where LDA exceeded 2.0. At the phylum level, *Firmicutes* was enriched in the Le group and *Bacteroidetes* and *Fusobacteria* were enriched in the Me group. At the family level, *Enterococcaceae* was enriched in the He group, *ACK M1* was enriched in the Le group and *S24 7*, *Fusobacteriaceae*, *Bacteroidaceae* and *Ruminococcaceae* were enriched in the Me group.

At the genus level, *Megamonas*, *Dolichospermum*, *Peptostreptococcus* and *Eubacterium* were enriched in the He group and *Bacteroides*, *Rothia* and *Faecalibacterium* were enriched in the Me group, as shown in Figure 6B,C. Heat maps at the species level further revealed the influences of the diet energy level on the fecal microbiota structure, as in Figure 6D. Compared with the Me group, the relative abundances of *Lactobacillus helveticus*, *Bifidobacterium pseudolongum*, *Lactobacillus vaginalis* and *Clostridium spiroforme* in both the Le and ME groups, and *Clostridium hiranonis*, *Lactobacillus coleohominis*, *Clostridium perfringens*, *Eubacterium biforme*, *Collinsella stercoris* and *Ruminococcus gnavus* in the He group, were all significantly increased. The relative abundances of *Blautia producta*, *Bacteroides plebeius*, *Faecalibacterium prausnitzii*, *Lactobacillus pontis*, *Prevotella copri*, *Cetobacterium somerae* and *Lactobacillus salivarius* in both the Le and ME groups, and *Ruminococcus torques*, *Bifidobacterium animalis* and *Clostridium celatum* in the He group, were low.

Random forest analysis can identify the complex nonlinear dependence between variables to enable the more effective and accurate classification of intestinal flora samples from each group [56]. As shown in Figure 6E, the relative abundances of *Eubacterium biforme*, *Collinsella stercoris*, *Clostridium hiranonis*, *Lactobacillus helveticus*, *Clostridium perfringens*, *Clostridium ruminantium* and *Ruminococcus gnavus* significantly increased and became the dominant strains in the He group. The relative abundances of *Ruminococcus torques*, *Clostridium disporicum*, *Clostridium celatum*, *Lactobacillus vaginalis* and *Clostridium spiroforme* significantly increased and became the dominant strains of the Le group. Most of the strains above are harmful bacteria, and the relative abundances of a variety of probiotics, such as *Faecalibacterium prausnitzii*, *Bacteroides plebeius*, *Blautia producta*, *Prevotella copri*, *Cetobacterium somerae* and *Lactobacillus pontis*, increased in the Me group. The above results show that adequate diet energy levels promoted the growth of probiotics and prevented the growth of harmful bacteria.

After examining the structural characteristics, the Picrust2 software was used to observe the influence of the fecal microbiota on metabolic pathways. As shown in Figure 6F, metabolic pathways in each group were concentrated in the aspects of metabolism and genetic information processing, among which the metabolism-related pathways accounted for the highest proportion of the total. The identification of bacterial functions between the Le and Me groups revealed that two distinctive pathways, including the metabolism of xenobiotics by cytochrome P450 (ko00980) and fluorobenzoate degradation (ko00364), were significantly represented, as shown in Figure 6G. There were no differential metabolic pathways between the He and Me groups.

### 3.6. Network Relation between Diet, Host Microbiota and Fecal Microbiota

To comprehensively evaluate our data and support a system-level understanding of the relation between diet, host and fecal microbiota, an integrative analysis of data from four dimensions was employed. We used four items in diet nutrition content, eight body condition indexes and nine serum biochemistry and fecal pH indexes in host phenotypic data, 10 phylum-level taxa and 20 genus-level taxa in fecal microbiota and eight fecal SCFAs, five primary BAs and 34 secondary BAs in fecal metabolites. 

**Figure 7:**
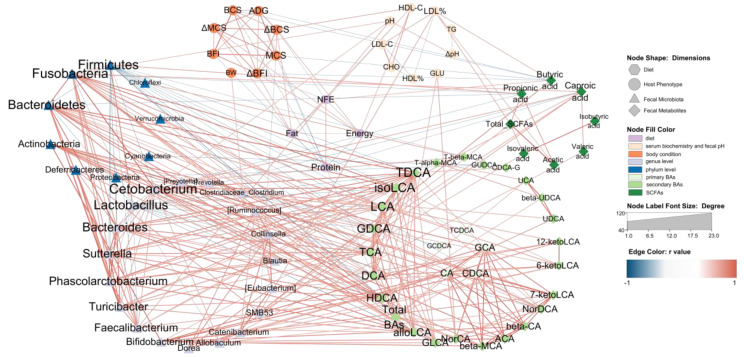
Network relation between diet, host and fecal microbiota. The network illustrates the interaction among diet, host and fecal microbiota. The hexagon nodes indicate the diet factor, the ellipse nodes indicate the host phenotype, the triangle nodes indicate the fecal microbiota, and the diamond nodes indicate fecal metabolites. The red edges indicate a positive correlation, and the blue edges indicate a negative correlation. The edges are the correlations with a Pearson’s correlation coefficient < −0.6 (or >0.6) and *p* < 0.05.

The Le, Me and He groups at T8 with 18 beagles were chosen for an association analysis. The Pearson correlation between all the data was evaluated. Data with strong correlation (−0.6 > r > 0.6 and *p* < 0.05) were used to construct an integrated visualization network containing eight circles. As shown in Figure 7, nine taxa were in the circle of the phylum level of the fecal microbiota, nineteen taxa were in the circle of the genus level of the fecal microbiota and twenty-five secondary BAs were in the fecal BAs circle. The diet nutrition content items, host phenotypic items, primary BAs and fecal SCFAs were all selected in the network and the results showed that secondary BAs, such as TDCA, isoLCA, LCA and GDCA et al., were strongly correlated with fecal microbiota, especially *Firmicutes*, *Fusobacteria*, *Bacteroidetes* and *Actinobacteria* at the phylum level, and *Cetobacterium*, *Bacteroides*, *Sutterella*, *Phascolarctobacterium*, *Turcibacter*, *Faecalibacterium*, *Bifidobacterium* and *Dorea* at the genus level. The taxa of these fecal microbiota all showed a positive correlation with secondary BAs, except *Firmicutes* and *Lactobacillus,* which were negatively correlated with BAs. It should be noted that primary BAs were mainly associated with five secondary BAs, including NorCA, bata-MCA, ACA, β-CA and 7-ketoLDA,d and were only correlated with *Bifidobacterium* at the genus level.

Different SCFAs showed different functions in the network. Butyric acid was negatively correlated with body condition indexes and positively correlated with the NFE content in the diet. Caproic acid was the only SCFA associated with BAs, such as GDCA and isoLCA. Most SCFAs are associated with *Fusobabacteria*, *Firmicutes*, *SMB53*, *Allobaculum* and LDL%. On the host phenotypic dimension, fecal pH showed a positive correlation with *Fusobacteria*, *Faecalibacterium* and *Cetobacterium*. LDL% was positively correlated with *SMB53*, butyric acid, acetic acid and total SCFAs. The GLU was the only serum biochemistry index associated with BAs. Diet dimension is mainly associated with body condition indexes and HDL-C, and it had no edge with the fecal microbiota dimension. Butyric acid, caproic acid and DCA were the only three fecal metabolites correlated with diet nutrition.

## 4. Discussion

Animals need energy for all their life activities, and energy comes from fat, protein and carbohydrates in the diet [57]. Changes in the composition of these components may alter the diet energy level and also affect the metabolism of the host, and fecal microbiota and related studies have been reported in humans [58,59], mice [60], pigs [61] and cattle [62]. In the current pet food market, brands and consumers tend to pay attention to the content and source of protein in pet food and usually ignore the energy content. Excessive or insufficient energy intake may have adverse effects on animals [63]. Research on the nutritional regulation of obesity in dogs and cats is increasing [64,65], but the question of how to ensure healthy weight loss in animals while controlling energy is currently an important topic in dog and cat nutrition research [1].

This study investigated the effects of diet energy factors on body condition, glycolipid metabolism, fecal microbiota and metabolites in adult beagles. It found that changing the energy level of the diet, while keeping the protein level constant, significantly affected the body condition of adult beagles. At the end of the eight-week trial, weight loss was observed in the Le group, whose dietary ME was 13.88 MJ/kg, while the Me (ME = 15.04 MJ/kg) and He (ME = 17.05 MJ/kg) groups did not show significant changes.

During feeding, it was found that dogs in the Le group could consume almost all of the 32 g/BW kg/d food, while dogs in the Me and He groups had varying degrees of leftovers, which may have been related to the fact that dogs can adjust their food intake according to their energy intake. A similar finding was obtained by Xue et al. [14], where adult beagle dogs lost 15 to 25% of their body weight after eight weeks of consuming a diet with ME of 13.54 or 12.93 MJ/kg diet, while no significant changes were observed after 24 weeks of consuming a dietary ME of 15.07 MJ/kg diet.

It is the nature of dogs to eat for energy. When the energy intake of a canine is satisfied, its appetite and consequently food intake decreases, which is regulated by the body’s hormones and the nervous system [66]. In a study by Hall et al. [67], it was found that dogs preferred balanced food with 25.2% protein, 15.8% fat and 44.9% carbohydrates over high-fat food with 24.6% protein, 28.4% fat and 44.9% carbohydrates, when the two diets had similar protein levels. The study also found that healthy adult dogs chose to consume most of their energy from fat (41%) and carbohydrates (36%) and there was a negative relation between fat and carbohydrate intake (r = −0.87). In view of the effects of the macronutrient composition of the diet on palatability and the appetite regulation mechanism of canines, it can be explained why there were no significant changes in body weight in the Me and He groups over eight weeks under adequate food conditions, and the body weights of the two groups were similar.

Compared with the other two groups, the BCS, MCS and BFI of the Le group showed significant decreases. The results of the Le group were mainly due to the imbalance of fat and carbohydrates and the lack of fat. The fat content of the Le diet was only 4.69%; it was lower than the NRC recommendation for adult dogs. The BCS is a common clinical scoring system to assess the body condition of dogs and cats, which can be used to determine the degree of obesity scoring. In this trial, the BCS of adult beagles increased with an adequate energy supply, while the Le group showed a decrease in BCS and entered a leaner body condition after being fed a low-energy diet for eight weeks. Many studies have shown that lowering the diet energy level by adjusting the macronutrient composition may reduce body weight and body condition [11,13,15,19,68]. These results were associated with inadequate energy intake, resulting in an inability to ensure the maintenance of energy requirements. The use of MCS for the clinical assessment of the nutritional status of dogs and cats is also an important indicator, where overweight animals may have significant muscle loss and animals with lower BCS may also have good muscle condition [69,70]. The BFI is used in the clinical assessment of the body fat percentage (BF%) by palpation to assess the risk of obesity [69]. In this trial, both the Me and He groups maintained good muscle mass and BF%, but dogs in the Le group showed a significant decrease in muscle mass and BF% after the eight-week trial. In combination with the BCS results, this suggests that if the energy supply is inadequate, the dogs will consume fat and protein to ensure maintenance energy requirements used for the most basic vital and voluntary activities. In one weight loss program study using a diet with ME = 12.39 MJ/kg, the protein content needed to be increased to 29% to ensure that no significant decrease in lean body mass or significant downregulation of fat mass and BF% occurred during the six-month weight loss period [13], but these results showed that dietary protein of 29% may be not enough for canine weight loss. Phungviwatnikul et al. [15] found a significant decrease in total body mass and lean mass in dogs fed a 42% protein, high-fiber diet with an ME of 11.76 MJ/kg for six weeks, suggesting that the content of dietary protein should be noted in the development of dog prescription diets for weight loss to avoid lean weight loss by reducing the diet energy level.

From the results of the network diagram, food factors were also associated with glycolipid metabolism, in addition to being more associated with body condition indexes. In this trial, the serum GLU of the Le and He groups was the highest at T4 and changed greatly over time, while the Me group was relatively stable. Changes in environmental temperature can cause changes in glucose metabolism in the body, as shown by changes in blood glucose [71], and diets with appropriate energy levels reduce this effect [72,73]. For serum biochemical indexes related to lipid metabolism, both the Le and He groups showed changes related to temporal factors, while the Me group was stable. The content of LDL-C in serum shows a significant positive correlation with the incidence of coronary heart disease, and it is an important index to evaluate the risk factors of individual coronary heart disease [74]. In canine obesity modeling and weight loss trials, high-fat diets were found to lead to a significant increase in serum CHO levels, while low-energy diets led to a decrease in CHO [13,14,15,75].

By analyzing the pH of the fresh feces of dogs, it was shown that the long-term consumption of either low- or high-energy diets caused fecal acidification, which was related to the metabolites in the feces and the composition of the gut microbiota, in several studies on canine nutrition [50,51,52,76,77,78]. Based on the above, the total SCFAs in the feces were measured in this study and were increased significantly in the Le group, especially for acetic acid and butyric acid. SCFAs are the end-products of carbohydrate metabolism by the gut flora, and the Le group had the highest carbohydrate content in the diet, explaining the above results. However, a significant decrease in SCFAs was also found in the He group compared to the beginning of the trial, which may be related to the lower carbohydrate and higher fat content in the diet. It has been shown that SCFAs can improve the inflammatory response of the body, induced by a high-fat diet [79,80], where butyric acid alleviates both oxidative stress and inflammation [81]. In contrast, SCFA deficiency leads to increased intestinal permeability, which triggers an inflammatory cascade response and induces the development of inflammatory diseases [82]. In addition, a sustained increase in glucagon-like peptide-1 (GLP-1) and PYY induced by propionate affects appetite regulation circuits in the brain and inhibits food intake [83,84]. In a study of pigs, both fecal acetic acid and butyric acid content were correlated with finishing weight [85].

Bile acids are involved in diet lipid absorption and utilization [86], and bile acids were analyzed that were closely related to lipid digestion and absorption in the intestine. The results showed that the profiles of BAs differed significantly between groups, and that the highest rise in total BAs was observed in the He group. Moreover, 7-ketolithocholic acid (7-ketoLCA), deoxycholic acid (DCA), norcholic acid (NorCA), allocholic acid (ACA) and 3β-cholic acid (β-CA) also showed similar trends with total BAs. Bile acids can be divided into two major groups according to the secretion pathway: one is primary bile acids directly secreted by the host, while the other is secondary bile acids after the modification of primary bile acids by intestinal flora. The bile acids, such as DCA mentioned above, are precisely secondary bile acids, which suggests that the digestion and utilization process of high-fat diets altered the metabolic activity of intestinal microbiota, reflected by similar results in humans [87,88]. A study in mice demonstrated that high-fat diets induced an increase in fecal DCA content and low-fat diets decreased the total BA content [89]. They also found that DCA disrupts intestinal mucosal barrier function by interfering with aryl hydrocarbon receptor (AHR) signaling in intestinal stem cells. The marker metabolites of bile acids in the feces of adult beagle dogs fed different energy level diets were significantly different. Decreased microbial secondary BA metabolites of DCA and HDCA were revealed in the feces of the low-energy group, and research showed that they were found responsible for stimulating 5-hydroxy tryptamine (5-HT) levels [90], involved in the regulation of appetite, weight and behavior [91]. Pan et al. [92] found that HDCA-treated mice exhibited reduced fat content and the metabolite HDCA was found to be significantly increased in the feces of uncoupling protein 1 (UCP1) knock-in pigs and had a negative relationship with backfat thickness. It is hypothesized that the decrease in fecal HDCA may be associated with energy deficiency in dog diets.

Changes in diet energy levels caused changes in intestinal metabolites, and there was an opposite trend in the changes in SCFAs and BAs between the Le and He groups. The reason may be the differences in energy levels in this experiment, which stemmed from the different carbohydrate and fat ratios, and the different intakes of carbohydrates and fat changed the structure and function of the intestinal flora—the network analysis confirmed this idea. The fecal microbiota compositions of the three groups were analyzed and it was found that changes in diet energy levels changed the flora structure. The change in diet energy level caused a decrease in microbiota diversity. In terms of flora composition, both temporal and energy level factors influenced the profiles of the genus and phylum levels and the number of ASVs. Similar findings have been obtained in canine studies on the relationship between food and the gut microbiota [93,94,95] and related studies have also demonstrated that *Fusobacterium* and *Clostridium* appear to be very strongly associated with the administration of raw-meat-based diets to cats and dogs [96].

The fecal microbiota results showed that either elevated or decreased diet energy levels inhibited the growth of probiotics and increased the growth of harmful bacteria. The relative abundances of *Faecalibacterium prausnitzii*, which is considered as one of the most important bacterial indicators of a healthy gut [97], significantly decreased in the Le and He groups. *Ruminococcus torques*, linked with gastrointestinal disease [98], was increased in the Le group. *Clostridium* was mainly associated with the Le and He groups and most of the associated strains were pathogenic or harmful [99,100]. *Bacteroides plebeius* improves muscle wasting in the chronic kidney [101], *Blautia producta* alleviates lipopolysaccharide-induced acute liver injury [102] and ameliorates high-fat-diet-induced hyperlipidemia [103], and *Cetobacterium somerae* and its metabolites are beneficial for liver health [104,105]. These probiotics were all enriched in the Me group. In terms of microbial function, a reduction in diet energy levels promoted the metabolism of xenobiotics by cytochrome P450 and fluorobenzoate degradation function. It has been found that the metabolism of xenobiotics via the cytochrome P450 pathway can be activated by increased energy restriction [106]. Fluorobenzoate degradation may be the key metabolic pathway involved in myasthenia gravis [107] and may be related to Crohn’s disease [108]. These facts suggest a potential risk associated with the prolonged feeding of low-fat, high-carbohydrate diets to adult beagles.

Diet–host–fecal microbiota interactions exist, and this relationship was also evident in the network analysis in this paper. Diets modify the body condition of dogs through their effect on body metabolism and act as a link between the host and the gut microbiota through secondary bile acids [109,110]. This suggests that further research is needed on the mechanism by which diet energy levels affect canine metabolism, and it suggests that attention should be paid to the role of fecal metabolites as biomarkers in pet food development to better monitor food–pet interactions.

## 5. Conclusions

We found that different diet energy levels altered the body condition, the glucolipid metabolism and the structure of fecal SCFAs, BAs and microbiota in adult beagles because of the interaction between diet, host and gut microbiota. Feeding dogs with a medium-energy-level diet with an ME of 15.04 MJ/kg was suitable for maintaining an ideal body condition and stable glucolipid metabolism and promoting the relative abundance of probiotics in the gut. Feeding low- (ME = 13.88 MJ/kg) or high-energy (ME = 17.05 MJ/kg) diets was harmful for glucostasis, decreased the diversity of the intestinal microbiota and promoted the relative abundance of pathogenic bacteria in the gut. Dogs fed a low-energy diet for an extended time may become lean and lose muscle mass, but a diet with low energy and 29% protein may not supply enough protein for dogs losing weight.

## Figures and Tables

**Table 1:** Ingredients and chemical compositions of experimental diets.

Item	Treatment		
	Le	Me	He
Ingredient	% as-is		
Corn	39.2	31.8	21.0
Corn gluten meal	7.0	8.1	9.2
Chicken fat	-	6.3	16.0
Broken rice	11	11	11
Beef meal	6.5	6.5	6.5
Bean pulp	6	6	6
Wheat shorts	5	5	5
Beet pulp	5	5	5
Poultry meal	5	5	5
Beef and bone meal	3	3	3
Vitamin and mineral premix	3	3	3
Liquid palatant	3	3	3
Hydrolyzed spray-dried chicken blood cells	2	2	2
Beer yeast powder	2	2	2
CaHPO₄	1.3	1.3	1.3
Chicken liver meal	1	1	1
Analyzed composition			
Dry matter (DM), %	93.85	93.89	94.25
	% DM		
Crude protein	29.99	28.93	29.06
Crude fat	4.69	10.22	19.74
Nitrogen-free extracts ^1^	53.38	48.91	39.40
Crude fiber	4.18	4.10	4.55
Ash	7.76	7.83	7.26
Calcium	1.04	1.08	1.02
Phosphorus	0.81	0.78	0.80
Lysine	1.29	1.18	1.21
GE, MJ/kg ^2^	18.28	19.46	21.23
ME, MJ/kg ^2^	13.88	15.04	17.05

^1^ Nitrogen-free extracts = 100 − (ash + crude protein + crude fat + crude fiber). ^2^ Gross energy (GE) was measured by bomb calorimetry; metabolizable energy (ME) = 8.5 kcal ME/g fat + 3.5 kcal ME/g protein + 3.5 kcal ME/g nitrogen-free extracts; 1 kcal = 4.184 kJ.

**Table 2:** Effects of diet energy levels on body weight (kg).

Time	Le	Me	He	SEM	*p*-Value
T0	12.50	12.48	12.58	0.49	0.997
T2	11.87	12.53	12.37	0.42	0.815
T4	11.30	12.24	12.36	0.39	0.500
T6	11.20	12.32	12.40	0.39	0.409
T8	11.27	12.68	12.68	0.38	0.233

**Table 3:** Changes in body weight and body condition of adult beagles.

Item ^1^	Energy Levels			SEM	*p*-Value		
	Le	Me	He		Energy	Liner	Quadratic
ADG (g/d)	−44.05 ^a^	7.14 ^b^	3.27 ^b^	8.68	0.016	0.015	0.084
ΔBCS ^2^	−0.25 ^a^	1.00 ^b^	1.67 ^b^	1.20	0.014	0.004	0.565
ΔMCS ^2^	−0.58 ^a^	0.25 ^ab^	0.83 ^b^	0.93	0.024	0.007	0.757
ΔBFI ^2^	−0.83 ^a^	6.67 ^ab^	12.50 ^b^	8.43	0.017	0.005	0.816

^1^ ADG = average daily gain; BCS = body condition score, MCS = muscle condition score and BFI = body fat index. ^2^ Changes in BCS, MCS and BFI between T8 and T0. ^a,b^ Within a row, means with different superscript letters were significantly different (*p* < 0.05).

**Table 4:** Effects of diet energy levels on serum glucose and lipid levels.

Item		GLU ^1^	CHO ^1^	TG ^1^	LDL-C ^1^	HDL-C ^1^	LDL-C% ^1^	HDL-C% ^1^
Unit		mmol/L	mmol/L	mmol/L	mmol/L	mmol/L	%	%
Reference Values [53]		3.61–6.55	3.50–6.99	0.20–1.30	-	-	-	-
T0	Le	4.89 ^AB^	5.44	0.62 ^A^	0.39	4.18	7.13 ^A^	77.03 ^B^
	Me	4.96	5.84	0.67	0.43	4.36	7.15 ^A^	75.80
	He	5.34 ^A^	5.44 ^A^	0.62	0.38 ^A^	4.28	6.82 ^A^	79.27 ^B^
T4	Le	5.59 ^B^	6.09	0.70 ^AB^	0.54	4.13	8.88 ^AB^	68.34 ^A^
	Me	5.64	7.01	0.69	0.66	4.60	9.28 ^B^	66.40
	He	6.41 ^B^	6.97 ^B^	0.84	0.62 ^B^	4.82	8.89 ^B^	69.45 ^A^
T8	Le	4.45 ^A^	5.43	0.88 ^B^	0.56	3.74 ^a^	10.50 ^B^	69.28 ^A^
	Me	4.69	6.85	0.65	0.73	4.28 ^ab^	10.17 ^B^	65.02
	He	4.95 ^A^	6.70 ^B^	0.72	0.62 ^B^	4.82 ^b^	9.21 ^B^	72.58 ^AB^
SEM		0.11	0.18	0.03	0.03	0.08	0.28	1.05
*p*-value	Time	<0.001	0.030	0.142	0.001	0.392	<0.001	<0.001
	Energy	0.058	0.085	0.591	0.317	0.005	0.662	0.192
	Time × Energy	0.840	0.755	0.217	0.935	0.286	0.915	0.923

^1^ GLU = glucose; CHO = total cholesterol; TG = triglyceride; LDL-C = low-density lipoprotein cholesterol; HDL-C = high-density lipoprotein cholesterol; LDL-C% = the ratio of LDL-C in CHO; HDL-C% = the ratio of HDL-C in CHO. ^A,B^ Within a column, means with different superscript capital letters were significantly different (*p* < 0.05) for the time factor in the same group. ^a,b^ Within a column, means with different superscript small letters were significantly different (*p* < 0.05) for the diet energy factor at the same time.

## Data Availability

The data presented in this study are available in the article.
